# Trifluoromethyl-substituted tetrathiafulvalenes

**DOI:** 10.3762/bjoc.11.73

**Published:** 2015-05-06

**Authors:** Olivier Jeannin, Frédéric Barrière, Marc Fourmigué

**Affiliations:** 1 Institut des Sciences Chimiques de Rennes, UMR 6226 CNRS-Université de Rennes I, Campus de Beaulieu, 35042 Rennes, France

**Keywords:** electrochemistry, electron withdrawing group (EWG), fluorine, tetrathiafulvalene (TTF)

## Abstract

A series of tetrathiafulvalenes functionalized with one or two trifluoromethyl electron-withdrawing groups (EWG) is obtained by phosphite coupling involving CF_3_-substituted 1,3-dithiole-2-one derivatives. The relative effects of the EWG such as CF_3_, CO_2_Me and CN on the TTF core were investigated from a combination of structural, electrochemical, spectrochemical and theoretical investigations. Electrochemical data confirm the good correlations between the first oxidation potential of the TTF derivatives and the σ_meta_ Hammet parameter, thus in the order CO_2_Me < CF_3_ < CN, indicating that, in any case, the mesomeric effect of the substituents is limited. Besides, crystal structure determinations show that the deformation of the unsymmetrically substituted dithiole rings, when bearing one, or two different EWG, and attributed to the mesomeric effect of ester or nitrile groups, is not notably modified or counter-balanced by the introduction of a neighboring trifluoromethyl group. DFT calculations confirm these observations and also show that the low energy HOMO–LUMO absorption band found in nitrile or ester-substituted TTFs is not found in TTF-CF_3_, where, as in TTF itself, the low energy absorption band is essentially attributable to a HOMO→LUMO + 1 transition. Despite relatively high oxidation potentials, these donor molecules with CF_3_ EWG can be involved in charge transfer complexes or cation radical salts, as reported here for the CF_3_-subsituted EDT-TTF donor molecule. A neutral charge transfer complex with TCNQ, (EDT-TTF-CF_3_)_2_(TCNQ) was isolated and characterized through alternated stacks of EDT-TTF-CF_3_ dimers and TCNQ in the solid state. A radical cation salt of EDT-TTF-CF_3_ is also obtained upon electrocrystallisation in the presence of the FeCl_4_^−^ anion. In this salt, formulated as (EDT-TTF-CF_3_)(FeCl_4_), the (EDT-TTF-CF_3_)^+•^ radical cations are associated two-by-two into centrosymmetric dyads with a strong pairing of the radical species in a singlet state.

## Introduction

**Scheme 1:**
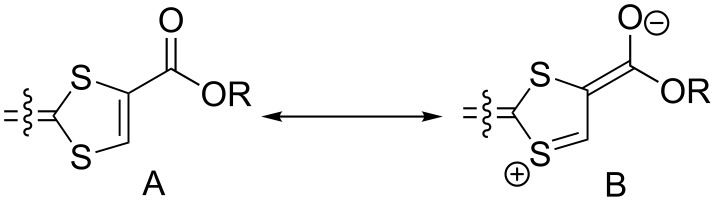
Mesomeric forms of 1,3-dithiole rings substituted with EWG.

Following three decades of extensive work toward the elaboration of conducting radical cation salts from tetrathiafulvalene (TTF) derivatives with electron-rich alkyl (tetramethyltetrathiafulvalene: TMTTF, tetramethyltetraselenafulvalene: TMTSF) or thioalkyl (ethylenedithiotetrathiafulvalene: EDT-TTF, bis(ethylenedithio)tetrathiafulvalene: BEDT-TTF) substituents [1], investigations of radical cation salts of tetrathiafulvalenes functionalized by electron-withdrawing groups (EWG) are less documented, essentially because the presence of such substituents as halogen, acyl, ester, amide or nitrile on the TTF redox core dramatically increases its oxidation potential and destabilizes the radical cation form. This strong anodic shift is particularly noticeable in tetrasubstituted TTFs such as TTF(CO_2_Me)_4_ [2–4], TTFCl_4_ [5], TTF(CF_3_)_4_ [2], or TTF(CN)_4_ [3], which oxidize into the radical cation at 0.80, 0.83, 1.05 or 1.12 V vs SCE respectively, to be compared with TTF itself which oxidizes at 0.33 V vs SCE. The associated instability of these radical species in moist air hindered in most cases their isolation in cation radical salts. This is all the more unfortunate since the electronegative atoms (O, N, Hal) within such EWG are expected to be able to engage, in the solid state at the organic–inorganic interface, in a variety of secondary non-bonding interactions such as hydrogen or halogen bonding [6–7], an issue of current strong interest in organic solid state chemistry [8–9]. However, it was also recognized that the introduction of only one or two of such EWG on the TTF core could limit this anodic shift, and accordingly, several tetrathiafulvalenes bearing only one or two ester [10], nitrile [11–14], amide [7,15–17], thioamide [18–20], or halogen [5] substituents were successfully engaged in radical cation salts by electrocrystallization, with intermolecular hydrogen [21–23] of halogen bond interactions [24–27]. Within such TTF derivatives, as reported by Bryce [28], an internal charge transfer (ICT) between the TTF and the EWG moieties increases the hydrophilicity of the TTF head groups and facilitates monolayer formation on the water surface for the preparation of Langmuir–Blodgett films. The structural and electronic properties of a series of ester [15], thioester [29–30], tertiary amide and thioamide [12] TTF derivatives have been then rationalized, based on: (i) the sizeable contribution of the mesomeric form B (Scheme 1) and, (ii) an ICT from the TTF-based HOMO to the EWG-based LUMO, also observed in primary and secondary amides [10]. Another consequence of the contribution of the B form is the shortening of the C–S bond opposite to the EWG, experimentally observed in the structures of such molecules.

**Scheme 2:**
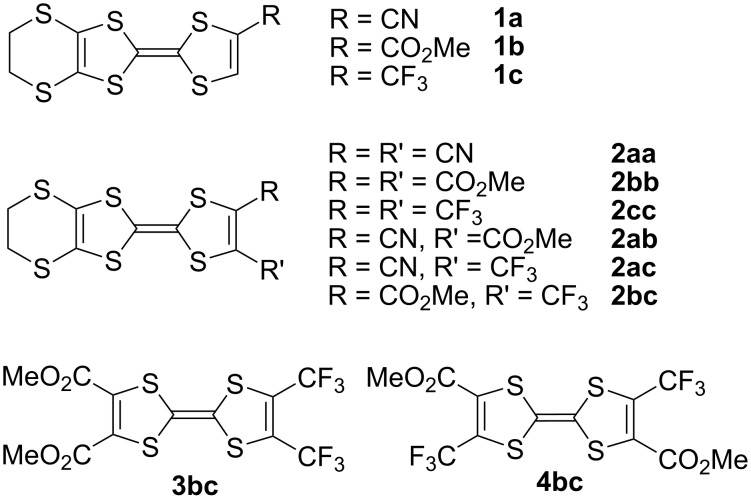
Investigated TTF derivatives bearing EWG.

More recently, we have reported another series of TTFs functionalized at various positions with the electron-withdrawing –CF_3_ (trifluoromethyl) group such as EDT-TTF(CF_3_) (**1c**) or EDT-TTF(CF_3_)_2_ (**2cc**) (Scheme 2) [31]. Single-crystal X-ray diffraction measurements revealed the recurrent formation of layered structures with a strong segregation of the fluorinated moieties and formation of fluorine bilayers [32–33], attributed to the amphiphilic character of those TTF derivatives upon CF_3_-functionalization. A strong anodic shift of the first oxidation potential was also noted for **1c** and **2cc**, when compared with the unsubstituted EDT-TTF molecule.

This work has been extended here to several novel disubstituted tetrathiafulvalenes bearing one CF_3_ group and one ester or nitrile group at the neighboring position as in **2ac** and **2bc** (Scheme 2). These derivatives offer an invaluable opportunity to evaluate the influence of the nature of the EWG on the structural and electronic properties of the TTF redox moiety, taking advantage of the marked differences between the three EWG now available: (i) the strongly electron withdrawing –CN group, (ii) the weaker –CO_2_Me group, both with important mesomeric effects, and, (iii) the –CF_3_ group, expected to exhibit essentially a strong −I inductive effect. Among the nine possible combinations **1** and **2** described above (Scheme 2), the unsymmetrically disubstituted **2ab** and **2ac** have not been reported to date. We describe here the syntheses of **2ac** from **2bc** and the single-crystal X-ray structure determinations of both **2ac** and **2bc** molecules. The preparation of the two positional isomers of bis(trifluoromethyl)-bis(carboxymethyl)tetrathiafulvalene **3bc** and **4bc** is also reported. The evolutions of (i) the geometry of the dithiole ring bearing the EWG, (ii) the electrochemical properties, (iii) the optical absorption (UV–vis) properties will be analyzed within the series, in order to evaluate the role of the CF_3_ group as electron-withdrawing substituent on the structural and electronic properties of the tetrathiafulvalene core, by comparison with that of the –CO_2_Me or –CN substituents. Furthermore, from the mono-substituted trifluoromethyl derivative **1c**, we were also able to isolate a charge transfer complex with TCNQ and a cation radical salt with FeCl_4_^−^. The structures of both compounds will be described, and the geometrical evolutions of the TTF core upon oxidation analyzed by comparison with the structure of neutral **1c**.

## Results and Discussion

### Syntheses

**Scheme 3:**
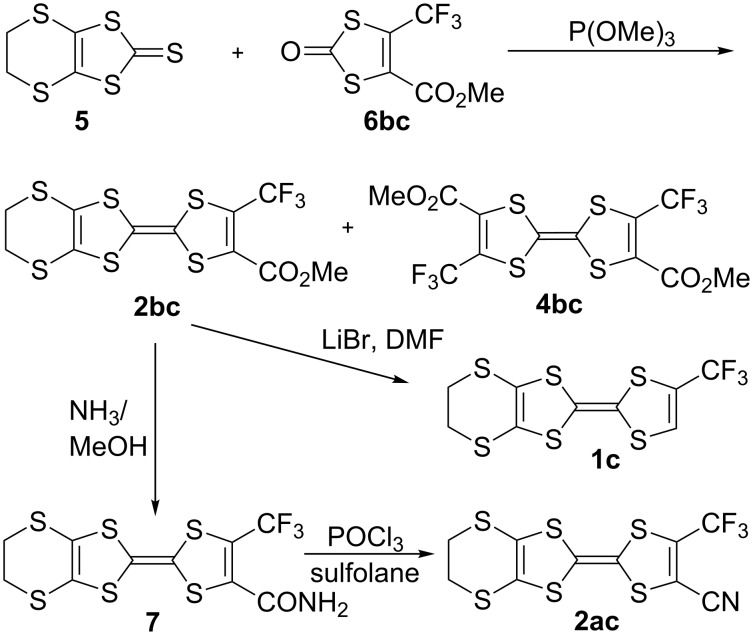
Synthetic procedures to the CF_3_-substituted **4bc**, **1c** and **2ac** molecules.

The reported preparation of **2bc** is based on the coupling reaction of the trithiocarbonate **5** with the dithiocarbonate **6bc**, affording also the symmetrical coupling product **4bc** (Scheme 3) [31]. Further decarboxylation of **2bc** with LiBr/DMF afforded **1c** while reaction with NH_3_ in MeOH gives the corresponding primary amide **7** in 63% yield. Its dehydration with POCl_3_ in sulfolane gives **2ac** in 60% yield.

**Scheme 4:**
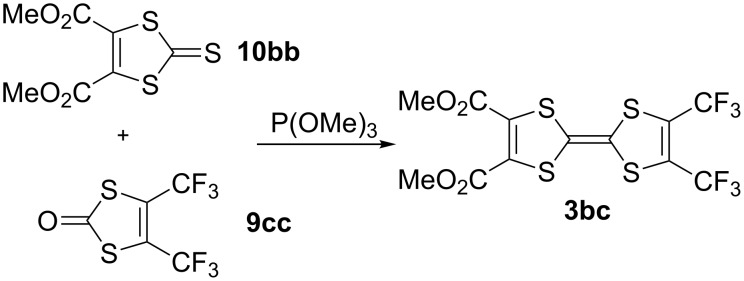
Synthetic procedure to **3bc**.

A similar phosphite-based cross-coupling reaction between the bis(trifluoromethyl)-1,3-dithiole-2-one derivative **9cc** and the diester derivative **10bb** gave the TTF **3bc** in 15% yield (Scheme 4).

With these extensive series at hand, it is now interesting to evaluate the role of the CF_3_ group as electron-withdrawing substituent on the structural and electronic properties of the tetrathiafulvalene core and to compare them with those of the –CO_2_Me or –CN substituents. This will be done here following two different approaches. First, we report the redox and optical absorption properties of the TTFs in solution, correlated with the electron-withdrawing character of the different substituents. Then, the relative effect of the three EWG on the solid state geometry of the dithiole ring will be described, based on the X-ray crystal structure analyses of four neutral TTF derivatives, i.e., the EDT-TTF derivatives **2ac** and **2bc** and the tetrasubstituted derivatives **3bc** and **4bc**.

### Redox properties

Cyclic voltammetry was used to evaluate the evolution of the donor strength with the nature and number of EWG. All derivatives exhibit two reversible oxidation waves. The *E*_1/2_ values for the EDT-TTF derivatives with one or two EWG are collected in Table 1. Compared with the unsubstituted EDT-TTF parent compound, redox potentials are shifted toward more anodic potentials with the introduction of the EWG with the following order CO_2_Me < CF_3_ ≤ CN. Note that this order is also confirmed by the evolution of the redox potentials of the tetrasubstituted TTF derivatives collected in Table 2.

**Table 1:** Electrochemical and spectroscopic data of the EDT-TTF-RR′ derivatives **1** and **2**. Reported values with other electrochemical references were converted to approximate values vs SCE and are given in italics. Potentials reported vs Fc^+^/Fc were converted to SCE by adding 0.39 V. Potentials reported vs Ag/AgCl were converted to SCE by adding −0.045 V. Lowest energy absorption maximum λ_max_ (nm) and molar extinction coefficient ε (L·mol^−1^·cm^−1^) are determined in CH_2_Cl_2_ unless otherwise specified).

RR′	solvent	reference electrode	*E*_1/2(ox1)_ (V)	*E*_1/2(ox2)_ (V)	references (electrochemistry)	Σσ_meta_	λ_max_ (ε)	references (UV–vis)

H, H	CH_3_CN	SCE	0.42	0.74	[34–35]	0.28	441 (451) 374 (1266, sh)	this work
H, CO_2_Me (**1b**)	CH_3_CN	SCE Ag/AgCl	*0.515* 0.56	*0.825* 0.87	[36]	0.63	480 418	[37]
H, CF_3_ (**1c**)	CH_2_Cl_2_	SCE Fc^+^/Fc	*0.56* 0.17	*0.99* 0.60	[17]	0.74	374 (2200)	this work
H, CN (**1a**)	PhCN	SCE	0.65	1.0	[9]	0.90	422 (725)	this work

CO_2_Me, CO_2_Me (**2bb**)	CH_3_CN	SCE Ag/AgCl	*0.595* 0.64	*0.905* 0.95	[20]	0.98	443 (1310)	this work
CF_3_, CO_2_Me (**2bc**)	CH_2_Cl_2_	SCE Fc^+^/Fc	*0.63* 0.24	*0.94* 0.64	[17]	1.09	467 (10800)	this work
CF_3_, CF_3_ (**2cc**)	CH_2_Cl_2_	SCE Fc^+^/Fc	*0.76* 0.37	*1.15* 0.76	[17]	1.2	422 (990)	this work
CN, CF_3_ (**2ac**)	CH_2_Cl_2_	SCE Fc^+^/Fc	*0.745* 0.355	*1.143* 0.753	this work	1.36	464 (760)	this work
CN, CN (**2aa**)			—	—	—	1.52	500 (464)	[11]

**Table 2:** Electrochemical and spectroscopic data of tetrasubstituted TTF derivatives. *E*_1/2_ values are reported in V vs SCE reference electrode. Lowest energy absorption maximum λ_max_ (nm) and molar extinction coefficient ε (L·mol^−1^·cm^−1^) are determined in CH_2_Cl_2_ unless otherwise specified).

	solvent	*E*_1/2(ox1)_	*E*_1/2(ox2)_	references (electrochemistry)	Σσ_meta_	λ_max_ (ε)	references (UV–vis)

TTF	CH_3_CN	0.33	0.71	[22]	0	446 (263)^a^	[38]
TTF(CO_2_Me)_4_ (**3bb**)	CH_3_CN	0.83	1.10	[22]	1.4	445 (1930)	[2–3]
*o*-TTF(CO_2_Me)_2_(CF_3_)_2_ (**3bc**)	CH_2_Cl_2_	0.95	1.28	this work	1.62	437 (2430)	this work
*E*-TTF(CO_2_Me)_2_(CF_3_)_2_ (**4bc**)	CH_2_Cl_2_	0.90	1.23	this work	1.62	467 (2280)	this work
TFF(CF_3_)_4_ (**3cc**)	CH_3_CN	1.05	1.28	[2]	1.84	416 (1390)	this work
TTF(CN)_4_ (**3aa**)	CH_3_CN	1.12	1.22	[3]	2.48	502 (2000)	[3]

^a^In CH_3_CN.

**Figure 1:**
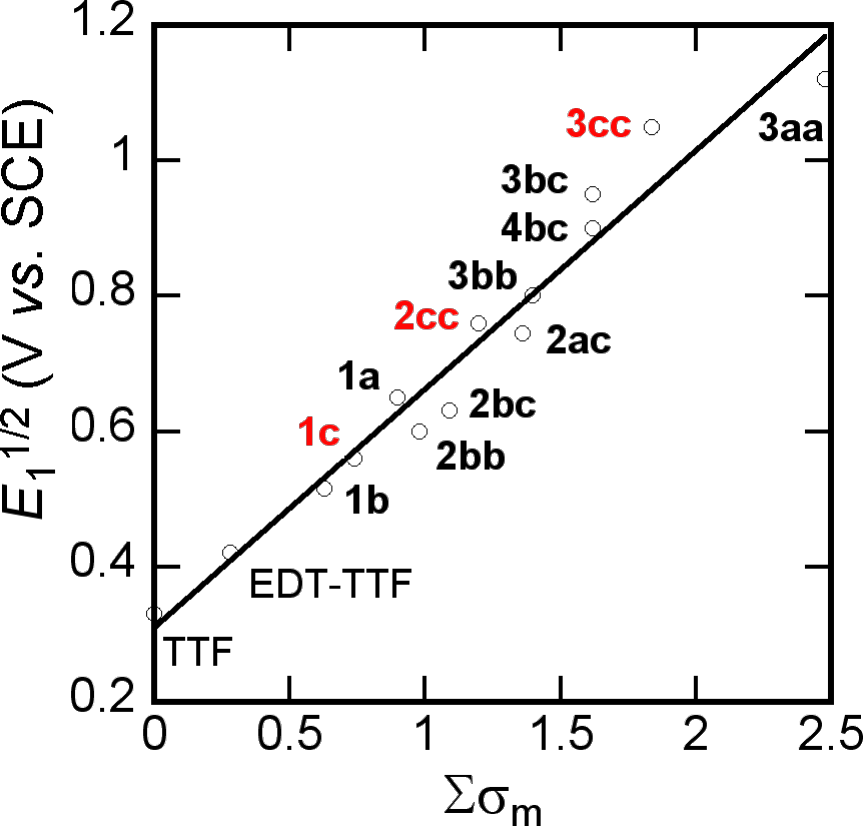
Correlation between the first oxidation potential *E*_1/2_ and the sum of the Hammet σ_meta_ parameters. TTF that only contain only CF_3_ EWG are given in red.

Earlier electrochemical investigations of various tetrathiafulvalene derivatives have shown that the best correlations between the first oxidation potential and the Hammet parameters [39–41] were actually found with the σ_meta_ constant of each substituent on the TTF core [20,42], indicating that, in any case, the mesomeric effect of the substituents was small. A similar satisfactory correlation with all TTF derivatives described here is shown in Figure 1 and demonstrates that the trifluoromethyl group anodic shift observed in the order CO_2_Me < CF_3_ < CN correlate well with the σ_meta_ Hammet constant.

### Optical properties

**Figure 2:**
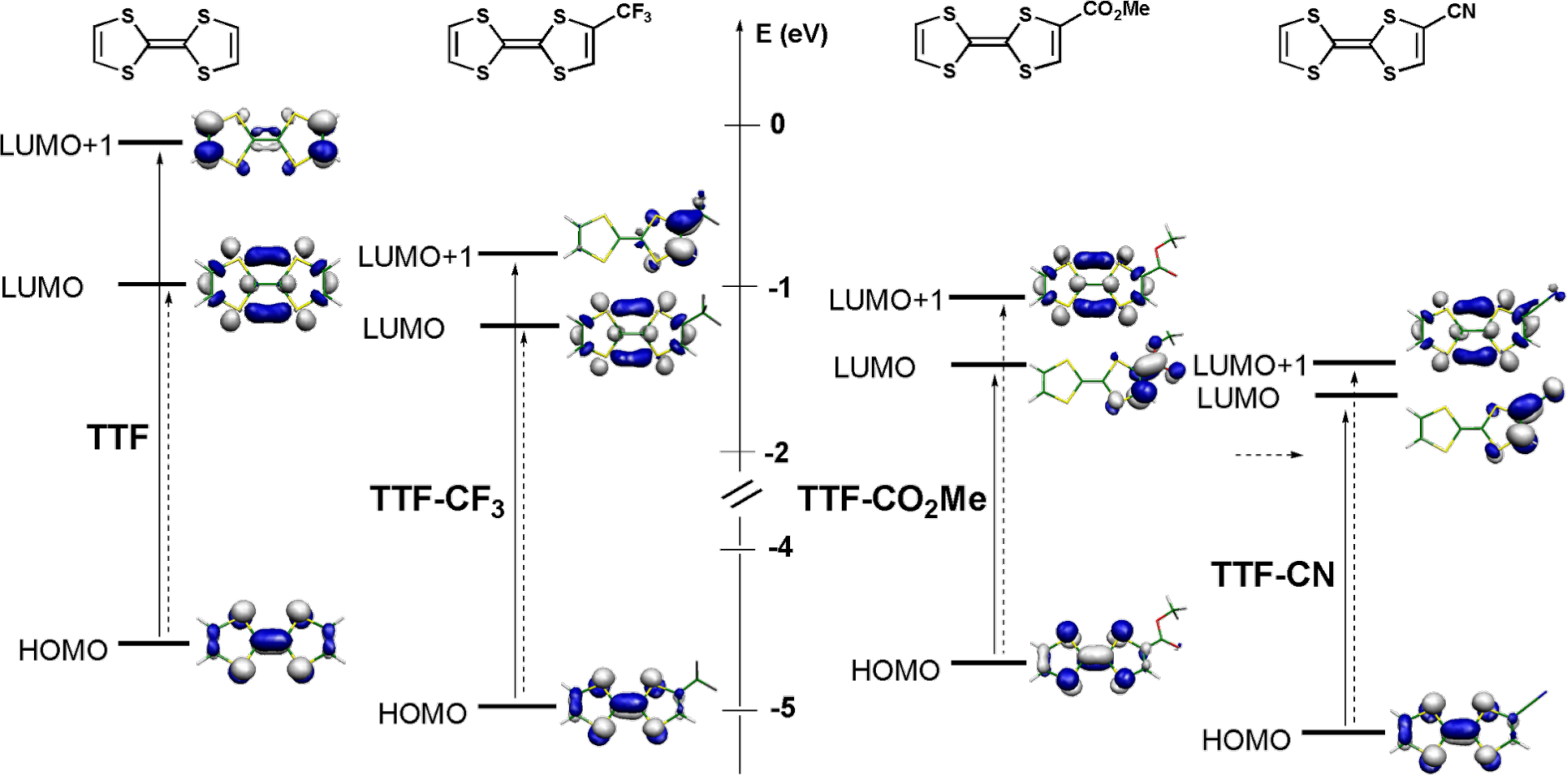
Calculated frontier orbitals of geometry-optimized [B3LYP/6-31G(d)] model compounds TTF, TTF-CF_3_, TTF-CO_2_Me and TTF-CN, with the lowest energy optical transitions deduced from TD-DFT calculations (see Table 3).

The evolution of the lowest energy absorption bands is also reported for the different TTF derivatives in Table 1 and Table 2. We note that the introduction of the trifluoromethyl group induces a blue shift of these absorptions, by comparison with EWG such as CO_2_Me or CN which move the absorption bands toward lower energies This point is actually correlated to the observed color difference, as the trifluoromethyl-substituted TFF derivatives are lightly orange colored, while the ester and cyano TTFs are dark red compounds. In order to rationalize these evolutions, we have performed TD-DFT calculations on the model molecules TTF, TTF-CF_3_, TTF-CO_2_Me and TTF-CN. The results are shown in Figure 2 and collected in Table 3, where a good correlation is found with the observed absorption bands experimentally observed in the four EDT-TTF derivatives, namely EDT-TTF, EDT-TTF–CF_3_ (**1c**), EDT-TTF–CO_2_Me (**1b**) and EDT–TTF-CN (**1a**).

**Table 3:** Calculated (TD-DFT, B3LYP/6-311G**) optical transitions in the model compounds TTF, TTF–CF_3_, TTF–CO_2_Me and TTF–CN, and comparison with the experimentally determined values in the analogous EDT-TTF derivatives.

model	transition	oscillator strength *f*	λ_calc_ (nm)	compound	λ_obs_ (nm)

TTF	HOMO→LUMO	0.0000	458.14	EDT-TTF	441 (451)
	HOMO→LUMO + 1^a^	0.0179	361.54		374 (1266, sh)

TTF–CF_3_	HOMO→LUMO	0.0009	448.62	**1c**	—
	HOMO→LUMO + 1^a^	0.0203	387.41		374 (2200)

TTF–CO_2_Me	HOMO→LUMO	0.0390	471.16	**1b**	480, see [35]
	HOMO→LUMO + 1	0.0001	450.83		418, see [35]

TTF–CN	HOMO→LUMO HOMO→LUMO + 1	0.0018	458.46	**1a**	422 (725)
	HOMO→LUMO HOMO→LUMO + 1	0.0263	452.63		
	HOMO→LUMO + 2^a^	0.0000	333.71		–

^a^The HOMO→LUMO + 4 is also involved in this transition. Cf Supporting Information File 1 for complete TD-DFT calculations.

Several points need to be emphasized. In pristine TTF as in TTF–CF_3_, the strongest, low energy transition is not the HOMO→LUMO transition but the HOMO→LUMO + 1 transition. Indeed, the LUMO in both molecules has a σ character while the LUMO + 1 has a π character. By contrast, the ester and cyano groups (−M EWG) are strongly conjugated with the π system to such a point that the order of the two lowest unoccupied orbitals is inverted. This inversion, with now a LUMO of π character, allows for a direct HOMO→LUMO optical transition in the two latter compounds. Besides, the −I inductive effect of the trifluoromethyl group stalilizes HOMO, LUMO and LUMO + 1 of TTF. As a consequence, its lower energy absorptions are only slightly shifted by comparison with TTF, as experimentally observed. On the other hand, the strong stabilization of the LUMO in the ester- or cyano-substituted TTFs leads to a large red shift of the low energy absorption of these molecules. Note also that the relative energy of the HOMO of the four different model TTFs is well correlated with the ranking deduced from the electrochemical measurements. The stabilization of the HOMO, associated with the anodic shift of the first oxidation potential is indeed strongest with the cyano group, with the following ordering H < CO_2_Me < CF_3_ < CN, as discussed above (Table 1 and Table 2).

### X-ray crystal structures of the neutral donor molecules

**Figure 3:**
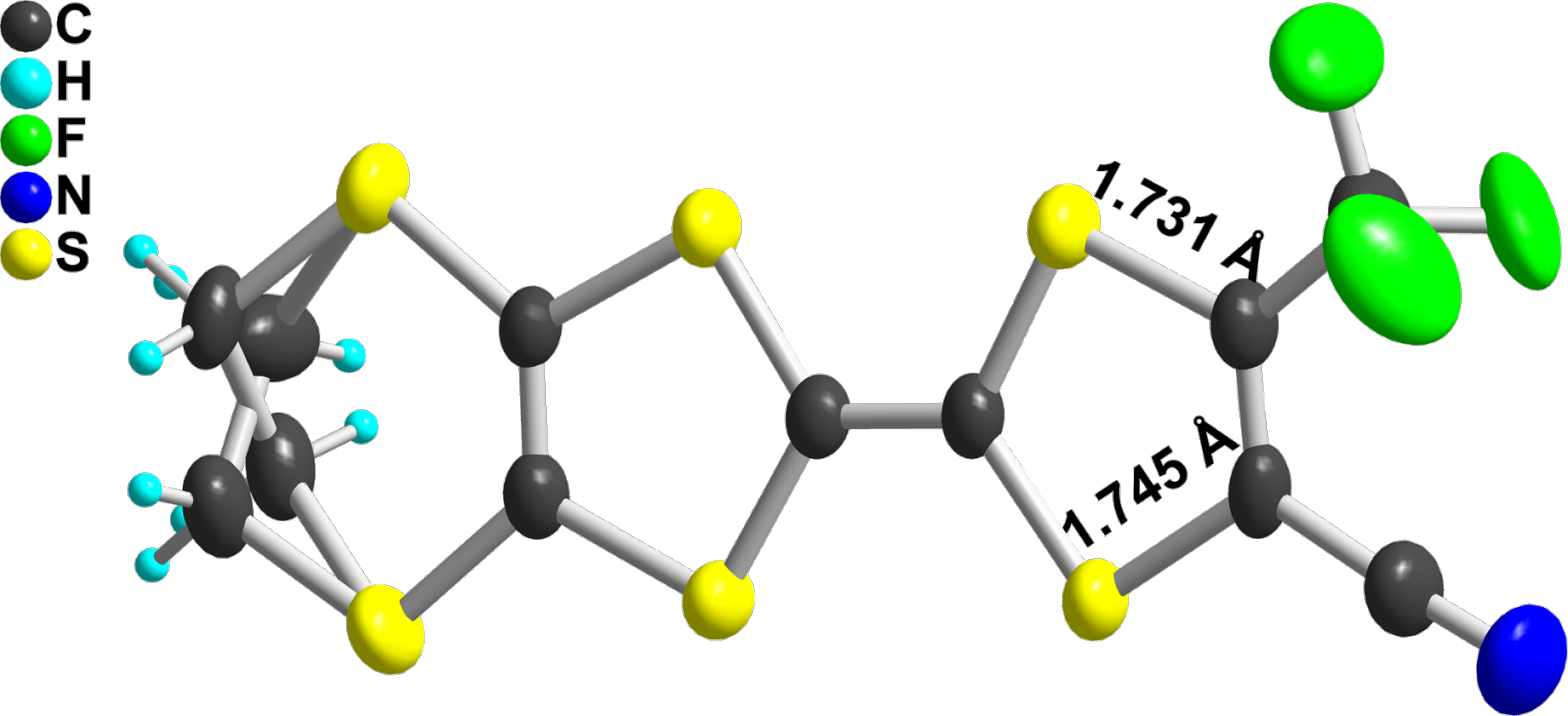
View of the **2ac** molecule. Thermal ellipsoids are shown at the 50% probability level.

**Figure 4:**
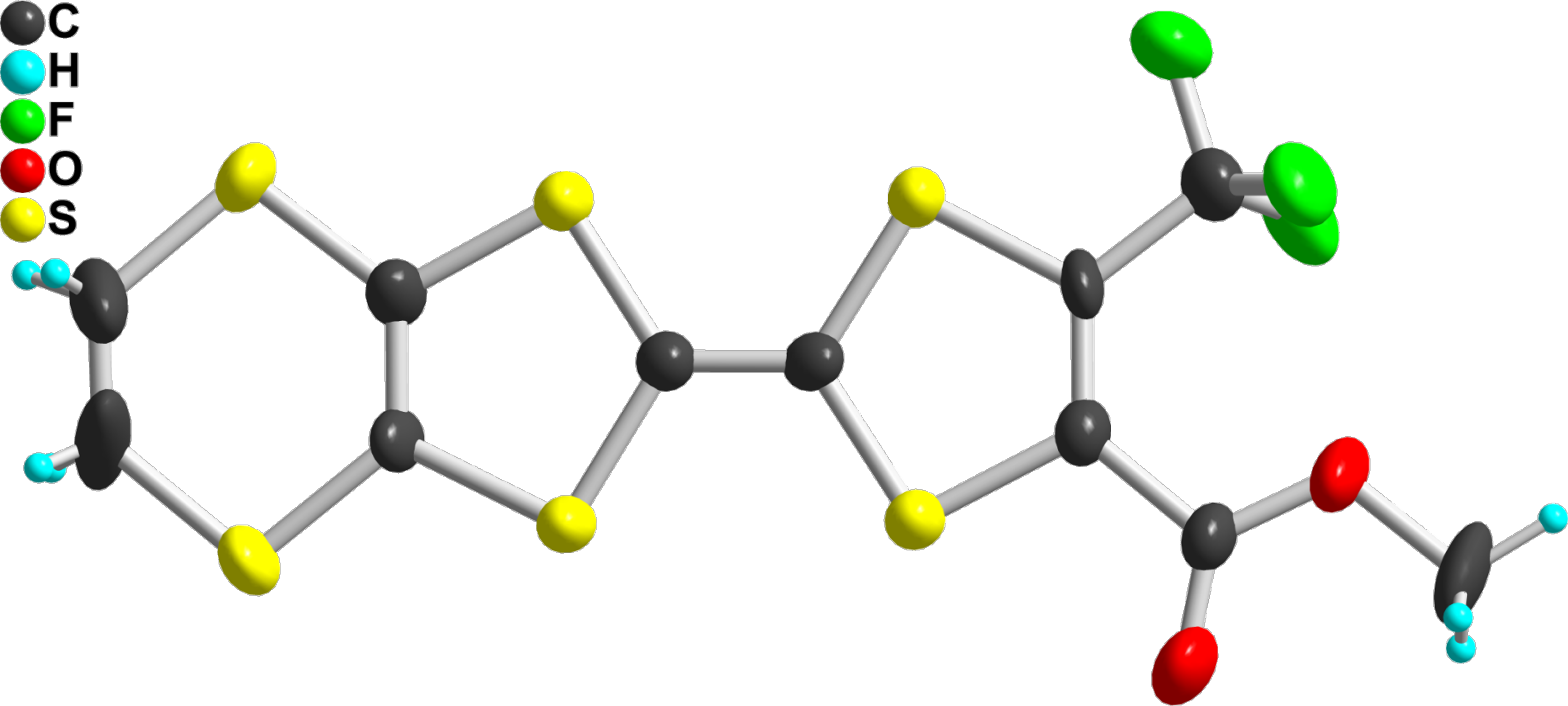
View of the **2bc** molecule. Thermal ellipsoids are shown at the 50% probability level.

As mentioned in the Introduction, the substitution of one hydrogen atom on the TTF core by one EWG such as ester or cyano group is known to distort the dithiole ring, as illustrated in Scheme 1. In the following, we want to evaluate the extent of this effect in the case of the trifluoromethyl group, and its evolution in competitive situations where two different EWG are on the same dithiole ring. For that purpose, we could obtain good quality crystals of the trifluoromethyl-substituted EDT-TTF derivatives with either one ester (in **2ac**) or one cyano group (in **2bc**) in ortho position to the CF_3_ group. **2ac** crystallizes in the monoclinic system, space group *P*2_1_, with one molecule in general position in the unit cell (Figure 3), affected by disorder on the ethylene bridge. On the other hand, **2bc** crystallizes in the triclinic system, space group *P*−1, with one molecule in general position in the unit cell (Figure 4). The ester group is coplanar with the TTF core and adopts a s-*trans* conformation. In both compounds, the CF_3_ group is not disordered as usually observed but adopts a fixed conformation with one fluorine atoms in the TTF mean plane, away from the other substituent. Bond lengths and angles are in the expected range. A dissymmetry of the C–S bonds (*b*, *b*′ in Table 4) in the dithiole ring bearing the different EWG is observed, with in both cases a shortening of the C−S bond close to the CF_3_ group. This polarization of the dithiole ring bearing one such EWG has been rationalized by Bryce on the basis of a sizeable contribution of a zwitterionic mesomeric form due to the influence of the EWG of −M character (Scheme 1) [8,17–18]. The fact that in both **2ac** and **2bc** molecules, this shortening affects the C−S bond closest to the CF_3_ group demonstrates unambiguously that the mesomeric electron-withdrawing effect of the CN or CO_2_Me groups is indeed stronger than that of CF_3_.

**Table 4:** Evolution of bonds distances within the dithiole ring in EDT-TTF derivatives substituted with one or two EWG. Definition of the C–S *b* and *b*′ bond distances are given in the scheme below.

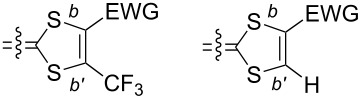				

compound	*b*	*b*′	100(*b*′ − *b*)/*b*′	references

CN, CF_3_ (**2ac**)	1.745(5)	1.731(9)	−0.8%	this work
CO_2_Me, CF_3_ (**2bc**)	1.727(23)	1.715(33)	−0.7%	this work
*E*-TTF(CF_3_)_2_(CO_2_Me)_2_ (**4bc**)	1.744(4) 1.742(4)	1.734(4) 1.732(4)	–0.6% –0.6%	this work

H, CN (**1a**)	1.760(7)	1.751(6)	−0.5%	[9]
H, CF_3_ (**1c**)	1.765(2)	1.737(2)	−1.6%	[17]

**Figure 5:**
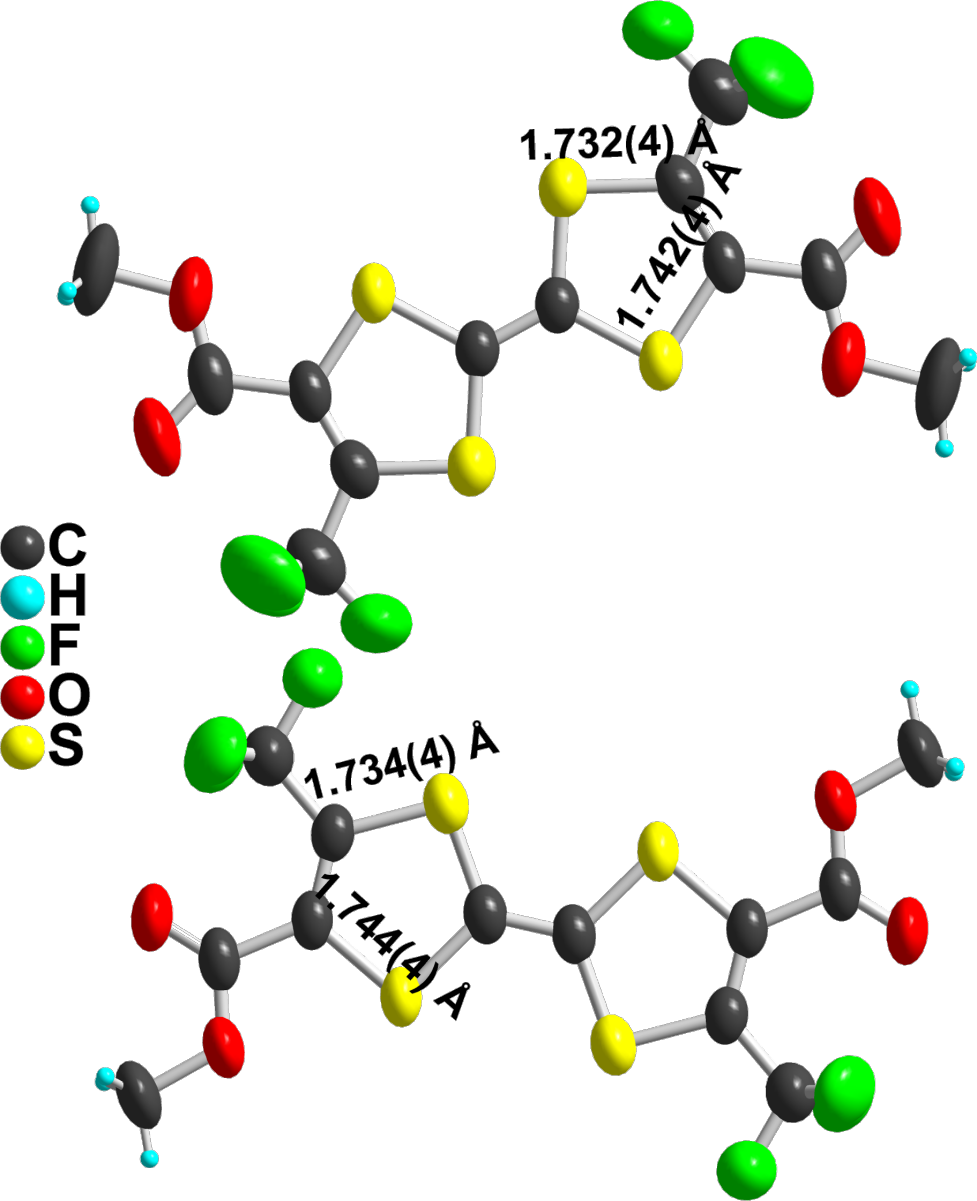
View of the two crystallographically independent **4bc** molecules. Thermal ellipsoids are shown at the 50% probability level.

A similar effect is also observed on the structure of the symmetrical TTF **4bc**, which was obtained as symmetrical coupling product of **6bc** during the preparation of **2bc** (Scheme 2). **4bc** crystallizes in the orthorhombic system, space group *Pbam*, with two crystallographically independent molecules, each of them located on a mirror plane and on an inversion center (Figure 5). In both molecules, a shorter C−S bond length is observed opposite to the CO_2_Me groups (Table 3), demonstrating again here that the (−M) mesomeric effect of the ester group exercises a stronger effect than the (−I) inductive effect of the CF_3_ group.

**Figure 6:**
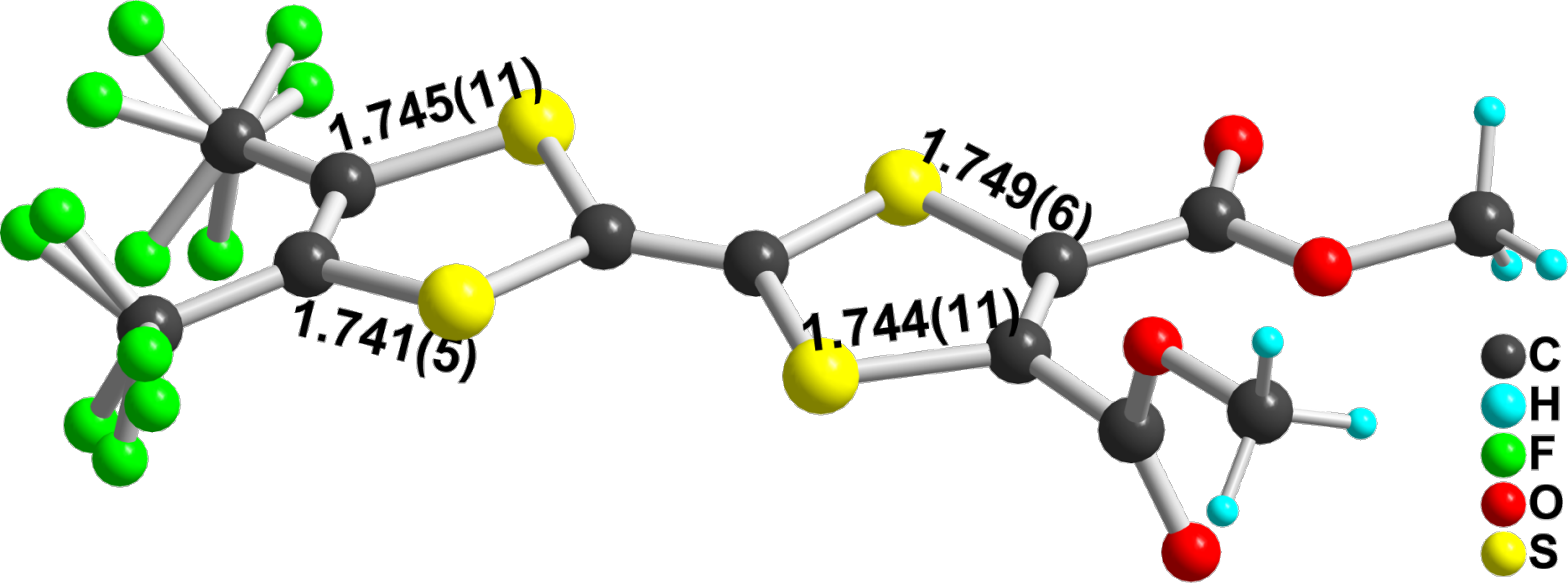
View of the **3bc** molecule. Note the disordered CF_3_ groups as well as the CO_2_Me group orthogonal to the TTF plane.

Another interesting insight is provided by the X-ray crystal structure of the other isomer **3bc**, where each dithiole ring is substituted with the same substituents, two CF_3_ or two CO_2_Me groups (Figure 6). We note first that the C–S bond distances (b) are now equal within the estimated standard deviations. We also observe that the localization of two CF_3_ moieties in ortho position to each other leads to a strong positional disorder of the fluorine atoms, at variance with the other structures described above. Furthermore, the two ester groups are not coplanar, one of them lies flat with the TTF core while the other one is almost perpendicular.

### Charge-transfer complex and radical cation salt

**Figure 7:**
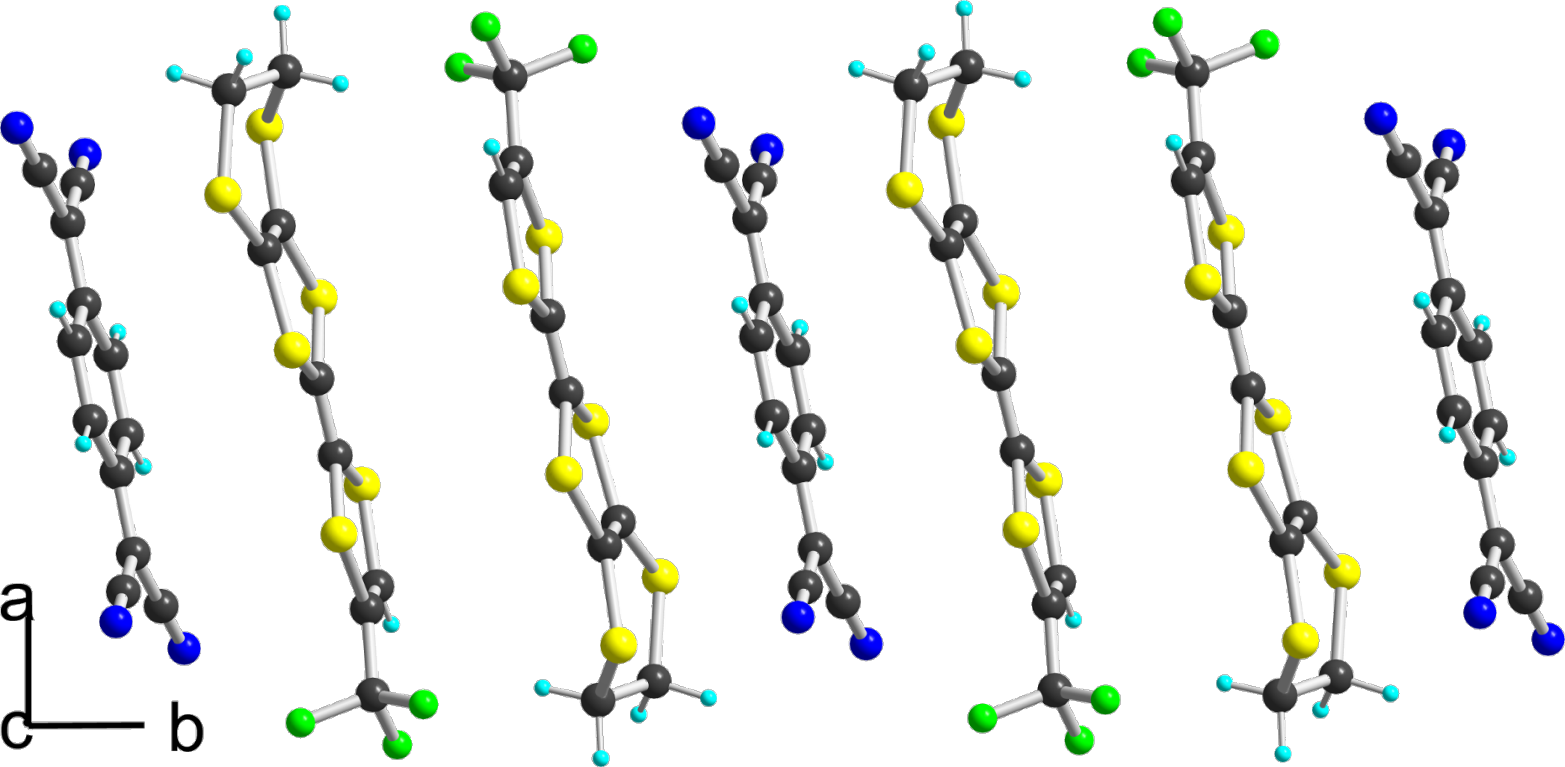
A view of the alternated stacks along the *b* axis in (**1c**)_2_(TCNQ).

The relatively low oxidation potential of the mono(trifluoromethyl) derivative **1c** (+0.21 V vs Fc^+^/Fc) prompted us to investigate the formation of cation radical salts upon chemical or electrochemical oxidation of **1c**. Indeed, treatment of a solution of **1c** with TCNQ afforded black, crystalline elongated plates which were analyzed to be a 2:1 complex, i.e., (**1c**)_2_(TCNQ). Electrocrystallization experiments were conducted with **1c** as well as **2cc** with a variety of anions, be they linear such as AuBr_2_^−^, I_3_^−^, ICl_2_^−^, tetrahedral (ReO_4_^−^, InBr_4_^−^) or octahedral (AsF_6_^−^). In most cases however, the electrochemically generated salts were extremely soluble, a consequence of the presence of the CF_3_ groups and there was no crystal growth on the anode. However, with **1c** as electroactive donor molecule and (*n*-Bu_4_N)(FeCl_4_) as electrolyte, layering of the CH_2_Cl_2_ solutions after electrolysis with pentane afforded crystals of a 1:1 phase formulated as (**1c**)(FeCl_4_).

(**1c**)_2_(TCNQ) crystallizes in the monoclinic system, space group *P*2_1_/*c*. One TCNQ molecule located on an inversion center and the EDT-TTF-CF_3_ molecule in general position in the unit cell generate molecular triads (**1c**)(TCNQ)(**1c**) which stack along the *b* axis (Figure 7).

The degree of charge-transfer within this system can be anticipated to be close to zero from the comparison of the redox potentials of EDT-TTF-CF_3_ (*E*_ox_^1/2^ = 0.21 V vs Fc^+^/Fc) and TCNQ (*E*_red_^1/2^ = −0.23 V vs Fc^+^/Fc, 0.17 V vs SCE) [43]. This is also confirmed from the intramolecular bond lengths (Table 5) within the central C_2_S_4_ core of the donor molecule, close to those observed in neutral EDT-TTF-CF_3_ (**2**) itself. The geometry of TCNQ can also give another evaluation of the degree of charge transfer. Based on the large number of reported TCNQ salts, three different correlations between the charge of the molecule and the bond lengths have been reported [44–46]. Applying those three correlations to the TCNQ bond lengths in (**1c**)_2_(TCNQ), averaged in *D*_2_*_h_* symmetry, gives calculated charges of −0.08, +0.11 and −0.16, confirming that we are here in presence of a neutral charge-transfer complex rather than a charge-transfer salt.

**Table 5:** Structural characteristics of the C_2_S_4_ central core in EDT-TTF-CF_3_ (**1c**) derivatives. ρ is the charge of **1c** in the different combinations. Bonds *a* (C=C) and *b*, *b*′, *c*, c′ (C_central_−S) are identified in the scheme below.

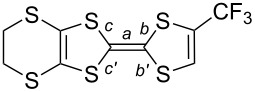							

	ρ	*a* (Å)	*b* (Å)	*b*′ (Å)	*c* (Å)	*c*′ (Å)	references

**1c**	0	1.348(3)	1.755(2)	1.758(2)	1.759(2)	1.759(2)	[17]
(**1c**)_2_TCNQ	≈0	1.336(4)	1.756(15)	1.763(6)	1.756(6)	1.755(15)	this work
(**1c**)(FeCl_4_)	≈1	1.382(6)	1.727(4)	1.733(4)	1.723(4)	1.708(4)	this work

**Figure 8:**
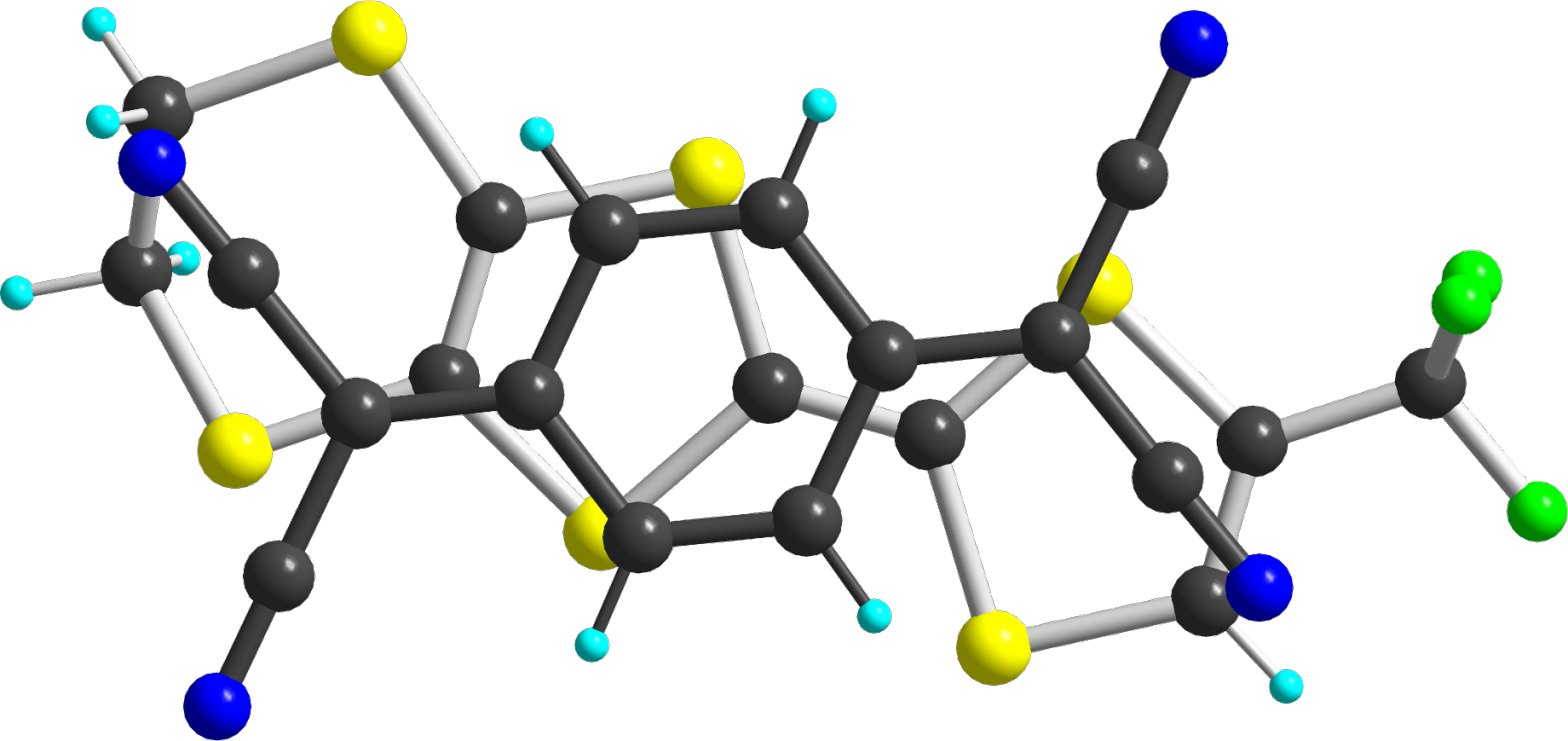
Detail of the overlap between donor and acceptor molecules in (**1c**)_2_(TCNQ).

Note also that this donor–acceptor interaction leads to a strong planarization of the dithiole rings of **1c** in (**1c**)_2_(TCNQ) with folding angles along the S^…^S hinge of the two dithiole rings amounting now to 10.13(17)° and 1.90(16)° on the dithioethylene and CF_3_ sides, respectively. By comparison, in the neutral donor molecule **1c**, the folding angles amount to 20.59(5) and 19.00(5)° respectively [31]. Such π–π interactions have been shown to derive from quadrupolar interactions between the π systems of both donor and acceptor moieties [47], and their geometrical characteristics to favor the strongest electrostatic interactions between the most electron-rich and electron-poor regions of both partners. In that respect, it appears here that the TCNQ acceptor essentially overlaps with the dithiole ring bearing the dithioethylene substituent, a likely consequence of the electron-withdrawing effect of the CF_3_ group on the other dithiole ring (Figure 8).

In the 1:1 salt of EDT-TTF-CF_3_ (**1c**) with FeCl_4_^−^, that is (**1c**^+•^)(FeCl_4_^−^), oxidation to the radical cation state strongly affects the central C_2_S_4_ core of the donor (Table 4) with a lengthening of the C=C central double bond *a*, and an associated shortening of the C_central_−S bonds *b*, *b*′, *c*, *c*′). Note that this effect is stronger on the (*c*, *c*′) C_central_–S bonds of the most electron-rich dithiole ring bearing the dithioethylene substituent.

**Figure 9:**
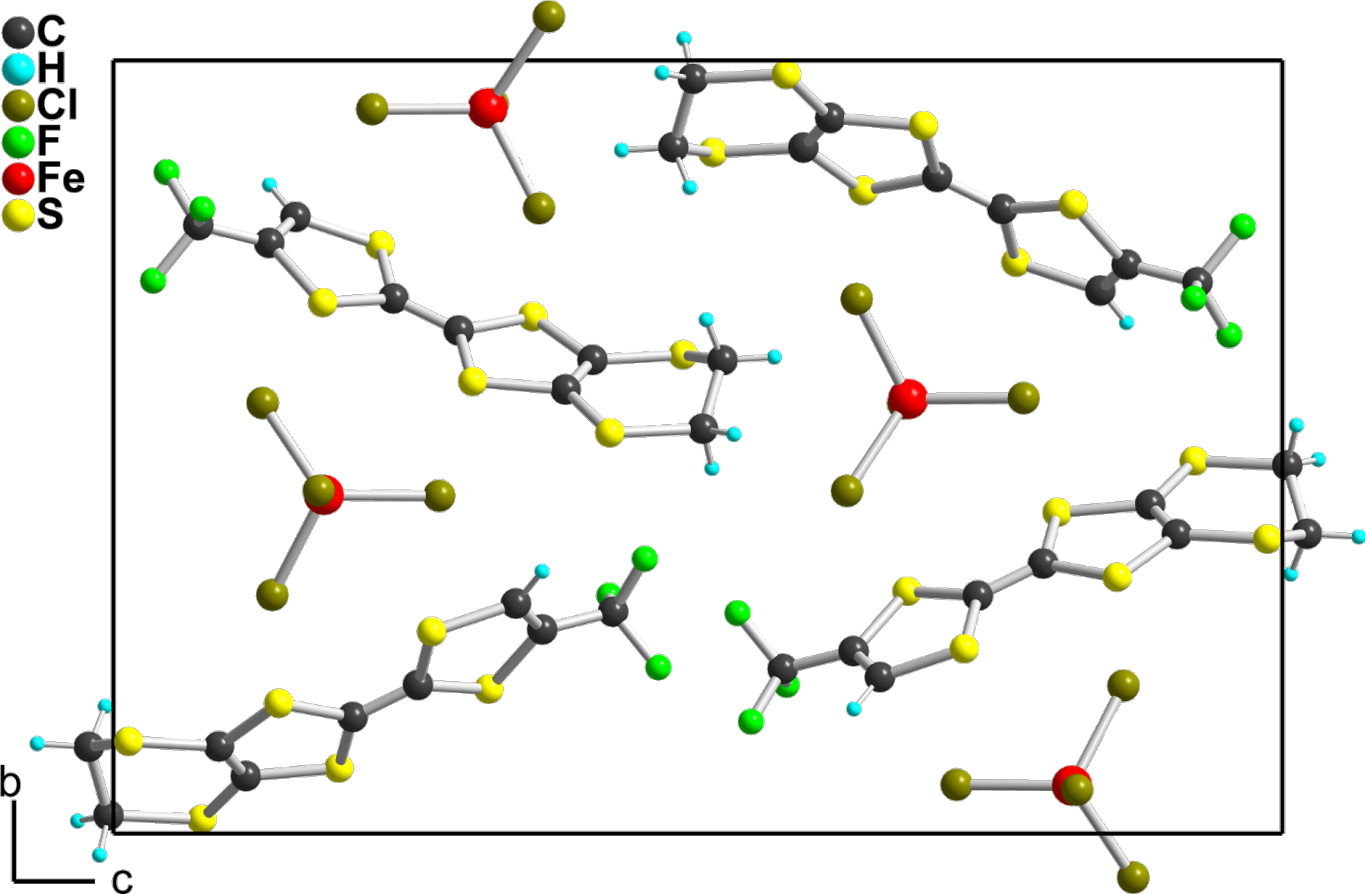
Projection view along the *a* axis of the unit cell of (**1c**^+•^)(FeCl_4_^−^).

In the solid state (Figure 9), the molecules are separated from each other by the FeCl_4_^−^ anions in the (*b*,*c*) plane. Along the *a* axis, they interact only laterally with long S^…^S intermolecular (>3.74 Å) into uniform spin chains. This solid-state arrangement is reminiscent of that observed with the analogous nitrile substituted EDT-TTF, that is EDT-TTF-CN, in the similar 1:1 (EDT-TTF-CN^+•^)(FeBr_4_^−^) salt [9], demonstrating that the CF_3_ moiety does not play here a crucial role in the solid state organization. Note that both charge transfer complex (**2**)_2_(TCNQ) and cation radical salt (**2**)(FeCl_4_) are expected to behave as insulators, because of zero charge transfer in the former and full charge-transfer in the latter. We were not able to determine the magnetic response of the FeCl_4_^−^ salt as the crystals are polluted with the starting electrolyte, due to the precipitation technique used to recover these highly soluble salts.

## Conclusion

Compared with other EWG such as –CN or –CO_2_Me, the CF_3_ substituent plays on the TTF electroactive core a peculiar role. From an electrochemical point of view, comparison of the relative role of the CN, CO_2_Me and CF_3_ EWG shows that, the electron-withdrawing nature of the CF_3_ moiety is intermediate between that of the CN and the CO_2_Me ones. On the other hand, the structural distortions introduced on the dithiole ring upon substitution with the CN and CO_2_Me (−M) EWG are not offset by the competing CF_3_ group, confirming its much weaker mesomeric effect on the conjugated dithiole core. Optical properties and theoretical calculations have shown that the HOMO–LUMO gap is not much modified in the CF_3_-substituted molecules, in sharp contrast with those TTF derivatives with CN or CO_2_Me EWG. In the latter indeed, the large stabilization of the LUMO localized on the EWG leads to a strongly decreased HOMO–LUMO gap associated with the well known dark color of these derivatives. Despite relatively high oxidation potentials, these donor molecules with CF_3_ EWG can be involved in charge transfer complexes or cation radical salts, as reported here for the CF_3_-substituted EDT-TTF donor molecule, in its 2:1 neutral CT complex with TCNQ, (**1c**)_2_(TCNQ) or its cation radical salt with FeCl_4_^−^. The high solubility brought by the trifluoromethyl substituent strongly limits the isolation of such salts by crystallization.

## Experimental

### General information

Commercially available reagents were used without further purification. Solvents were distilled under Ar. THF and Et_2_O were dried over KOH before distillation from Na/benzophenone. CH_3_CN and CH_2_Cl_2_ were distilled over P_2_O_5_ and MeOH over Mg/I_2_. Column chromatography was performed on silica gel. ^1^H, ^13^C and ^19^F NMR spectra were obtained on a Bruker Avance DRX500 spectrometer at 500.04 MHz for ^1^H, 470.28 MHz for ^19^F and 125.75 MHz for ^13^C. Chemical shifts were recorded in parts per million (ppm) downfield from tetramethylsilane (TMS). Coupling constants (*J*) are reported in Hz and refer to apparent peak multiplications. The abbreviations s and q stand for singlet and quartet. Elemental analyses were performed at the Service de Microanalyses, Institut de Chimie des Substances Naturelles (ISCN), Gif/Yvette (France). MALDI-TOF MS spectra were obtained from a Bruker Biflex-IIITM equipped with a 337 nm laser.

### Syntheses

**Preparation of EDT-TTF(CONH_2_)(CF_3_) (7):** EDT-TTF(CO_2_Me)(CF_3_) (**2bc**) [17] (0.2 g, 0.47 mmol) was added to a MeOH solution (20 mL) saturated with gaseous NH_3_. The resulting suspension was stirred for 90 min and filtered. The solid was recrystallized from CH_3_CN to afford **7** as red–burgundy needles (120 mg, 0.3 mmol). Yield: 63%; mp dec. 150–160 °C; ^1^H NMR (*d*_6_-acetone) δ 3.44 (s, 4H), 7.48 ppm (d, 2H); ^19^F NMR (CDCl_3_) δ −58.53 (s) ppm. Anal. calcd for C_10_H_6_F_3_NOS_6_: C, 29.62; H, 1.49; N, 3.45; found: C, 29.57; H, 1.34; N, 3.39; MS *m*/*z*: calcd, 404.87, found, 404.90.

**Preparation of EDT-TTF(CN)(CF_3_) (2ac):** A solution of EDT-TTF(CONH_2_)(CF_3_) (**7**) (0.3 g, 0.74 mmol) and POCl_3_ (0.2 mL, 2.15 mmol) in sulfolane (4 mL) is heated under stirring at 110 °C for 5 h. After cooling, the solution is poured in 100 mL of iced water and filtered. The dried precipitate is purified twice by column chromatography on silica gel with dichloromethane elution. Crystals were obtained by diffusion of pentane into a concentrated dichloromethane solution (0.17 g, 0.044 mmol). Yield: 60%; mp 176 °C (CH_2_Cl_2_/hexane); ^1^H NMR (CDCl_3_) δ 3.32 (s, 4H) ppm; ^13^C NMR (CDCl_3_) δ 30.10 (s), 104.58 (s), 107.86 (q, *J*^3^_CF_ = 3.84 Hz), 108.47 (s), 113.97 (s), 114.20 (s), 118.81 (q, *J*^1^_CF_ = 274.47 Hz), 118.30 (s), 138.43 (q, *J*^2^_CF_ = 36.47 Hz) ppm; ^19^F NMR (CDCl_3_) δ −59.20 (s) ppm; Anal. calcd for C_10_H_4_F_3_NS_6_: C, 30.99; H, 1.04; N, 3.61; found: C, 30.82; H, 1.01; N, 3.49; MS *m*/*z*: calcd, 386.86, found, 386.69.

**Preparation of *o*-TTF(CO_2_Me)_2_(CF_3_)_2 _(3bc):** A solution of bis(trifluoromethyl)-1,3-dithiole-2-thione (**9cc**) (2 g, 7 mmol) and bis(carbomethoxy)-1,3-dithiole-2-thione (**10bb**) (6 g, 24 mmol) in P(OMe)_3_ (15 mL) is heated at 110 °C for 16 h. After evaporation of the solvent under vacuum, the crude residue is purified twice by chromatography on silica gel with pentane/dichloromethane elution (50:50). The red fraction was collected and crystallized from CH_2_Cl_2_/hexane (0.51 g, 1.1 mmol). Yield: 15%; mp 75°C; ^1^H NMR (CDCl_3_) δ 3.86 (s, 6H) ppm; ^13^C NMR (CDCl_3_) δ 54.04 (s), 104.74 (s), 114.74 (s), 119.21 (q, *J*^1^_CF_ = 276 Hz) (s), 129.05 (q, *J*^2^_CF_ = 42 Hz), 132.41 (s), 159.83 (s) ppm; ^19^F NMR (CDCl_3_) δ −56.25 (s) ppm; Anal. calcd for C_12_H_6_F_6_O_4_S_4_: C, 31.58; H, 1.33; found: C, 31.79; H, 1.14; MS *m*/*z*: calcd, 455.91; found, 455.6.

**Preparation of (1c)_2_(TCNQ):** EDT-TTF-CH_3_ (**1c**) (20 mg, 5.5 × 10^−5^ mol) and TCNQ (5.6 mg, 2.75 × 10^−5^ mol) were dissolved in hot CH_3_CN (2 mL) and the mixture slowly cooled to room temperature. No crystal formation was observed at this stage. The solution is allowed to stand in a fridge for 15 days to produce thin needles which were filtered and recrystallized by slow evaporation in CH_3_CN to give the title compound as elongated black plates (20 mg, 78%); Anal. calcd for C_30_H_14_F_6_N_4_S_12_: C, 38.78; H, 1.52; N, 6.03; found: C, 38.61; H, 1.51; N, 6.08; IR ν_CN_ (KBr): 2222 cm^−1^.

**Preparation of (1c)(FeCl_4_):** The electrocrystallization of **1c** (11 mg) in a CH_2_Cl_2_ solution (15 mL) of (Et_4_N)(FeCl_4_) (208 mg) as electrolyte did not afford any crystals. Further layering of the solution recovered from the anodic compartment with pentane afforded dark crystals of a 1:1 phase formulated as (**1c**)(FeCl_4_), polluted with the yellow crystals of (Et_4_N)(FeCl_4_).

### Crystallography

Single crystals were mounted on the top of a thin glass fiber. Data were collected either on a Stoe-IPDS at room temperature or on a Nonius KappaCCD diffractometer at 150 K, both equipped with graphite-monochromated Mo Kα radiation (λ = 0.71073 Å). Structures were solved by direct methods (SHELXS-97) and refined (SHELXL-97) [48] by full-matrix least-squares methods, as implemented in the WinGX software package [49]. Absorption corrections were applied. Hydrogen atoms were introduced at calculated positions (riding model), included in structure factor calculations, and not refined. Crystallographic data are summarized in Table 6.

**Table 6:** Crystallographic data.

compound	**2ac**	**2bc**	**3bc**	**4bc**	(**1c**)_2_TCNQ	(**1c**)FeCl_4_

formula	C_10_H_4_F_3_NS_6_	C_11_H_7_F_3_O_3_S_6_	C_12_H_6_F_6_O_4_S_4_	C_12_H_6_F_6_O_4_S_4_	C_15_H_7_F_3_N_2_S_6_	C_9_H_5_Cl_4_F_3_FeS_6_
fw	387.50	420.53	456.41	456.41	464.59	560.14
cryst syst	monoclinic	triclinic	monoclinic	orthorhombic	monoclinic	orthorhombic
space group	*P*2_1_	*P*−1	*P*2_1_/*a*	*Pbam*	*P*2_1_/*c*	*P*2_1_2_1_2_1_
*a*/Å	5.0849(5)	5.1515(12)	7.4209(8)	28.000(3)	13.2146(15)	5.9348(5)
*b*/Å	10.9285(12)	11.998(2)	17.0659(17)	8.7129(9)	11.1448(8)	14.4165(15)
*c*/Å	12.7619(13)	12.974(3)	13.5707(15)	7.2091(7)	13.1662(15)	21.785(2)
α/deg	90	103.96(3)	90	90	90	90
β/deg	97.741(12)	90.02(3)	97.834(13)	90	106.997(13)	90
γ/deg	90	90.45(3)	90	90	90	90
*V*/Å^3^	702.72(13)	778.2(3)	1702.6(3)	1758.8(3)	1854.3(3)	1863.9(3)
*Z*	2	2	4	4	4	4
*d*_calc_/Mg m^−3^	1.831	1.795	1.781	1.724	1.664	1.996
diffractometer	Stoe-IPDS	Stoe-IPDS	Stoe-IPDS	Stoe-IPDS	Stoe-IPDS	KappaCCD
temp/K	293(2)	293(2)	293(2)	293(2)	293(2)	150(2)
μ/mm^−1^	0.991	0.910	0.636	0.616	0.768	2.072
θ-range/deg	2.45–25.75	1.75−25.94	1.85–25.85	2.45−25.98	2.44−25.83	2.34−26.02
measured refls	6805	7498	12937	10664	13683	38263
indep. refls	2682	2778	3263	1861	3518	3675
*R*_int_	0.0334	0.126	0.0639	0.0815	0.065	0.109
I > 2σ(I) refls	1986	989	2161	833	2190	2932
abs. corr.	multi-scan	none	gaussian	multi-scan	multi-scan	multi-scan
*T*_max_, *T*_min_	0.786, 0.851	—	0.782, 0.901	0.937, 0.927	0.912, 0.822	0.769,0.733
refined params	199	199	289	151	236	228
*R*(*F*), *I*>2σ(*I*)	0.0327	0.0442	0.0320	0.0342	0.0339	0.0410
*wR*(*F*^2^), all	0.0765	0.0909	0.0783	0.0654	0.0814	0.0538
res. Δρ (e Å^−3^)	+0.297, −0.357	+0.29, −0.28	0.211, −0.166	+0.18, −0.16	+0.32, −0.20	+0.41, −0.42

### Computational details

DFT [50–51] calculations were performed with the hybrid Becke-3 parameter exchange functional [52–54] and the Lee–Yang–Parr nonlocal correlation functional [55] (B3LYP) implemented in the Gaussian 03 (revision D.02) program suite [56], using the 6-31G(d) basis set and a quadratically convergent self-consistent field procedure with the default convergence criteria implemented in the program. The X-ray diffraction data of model compounds were used as a starting point for initial geometry optimization calculations. Final geometries are given in Supporting Information File 1. Representation of frontier orbitals included in Figure 2 were generated with Molekel 4.3 [57]. TD-DFT calculations were performed at the B3LYP/6-311G** level of theory, on the previously converged geometries.

## Supporting Information

File 1 Optimized geometries of the model molecules TTF, TTF–CN, TTF–CF_3_ and TTF–CO_2_Me and results of the TD-DFT calculations.

File 2 X-ray data for the reported structures.
