# Supplementary material for: Trifluoromethyl-substituted tetrathiafulvalenes
Source: Beilstein J Org Chem. 2015 May 6;11:647–58. doi: 10.3762/bjoc.11.73 (PMC4464086; doi:10.3762/bjoc.11.73)
Supplement: File 1 — Optimized geometries of the model molecules TTF, TTF–CN, TTF–CF3 and TTF–CO2Me and results of the TD-DFT calculations. [file Beilstein_J_Org_Chem-11-647-s001.pdf]

Supporting Information

for

Trifluoromethyl-substituted tetrathiafulvalenes

Olivier Jeannin, Frédéric Barrière and Marc Fourmigué*

Address: Institut des Sciences Chimiques de Rennes, UMR 6226 CNRS-Université de Rennes I, Campus de Beaulieu, 35042 Rennes, France

Email: Marc Formigué - marc.fourmigue@univ-rennes1.fr

* Corresponding author

Optimized geometries of the model molecules TTF, TTF–CN, TTF–CF_3 and TTF–CO_2Me and results of the TD-DFT calculations

TTF

Optimized geometry

C	0	0.04201	0.05705	0.00807
C	0	0.04248	0.05622	1.34516
S	0	1.58895	-0.12695	2.17271
C	0	2.56465	-0.0755	0.67564
S	0	1.58789	-0.12511	-0.8208
C	0	3.91307	-0.01055	0.6752
S	0	4.89017	0.03206	2.17163
C	0	6.41134	0.36298	1.34309
C	0	6.41086	0.36381	0.006
S	0	4.88911	0.0339	-0.82187
H	0	-0.84376	0.14133	-0.61065
H	0	-0.84285	0.13974	1.96461
H	0	7.28494	0.53122	1.96197
H	0	7.28403	0.53281	-0.61329

TD-DFT calculations

```
Excitation energies and oscillator strengths:

 Excited State   1:   Singlet-A      2.7062 eV  458.14 nm  f=0.0000
      52 -> 53         0.69382
 This state for optimization and/or second-order correction.
 Copying the excited state density for this state as the 1-particle
RhoCI density.

 Excited State   2:   Singlet-A      3.4293 eV  361.54 nm  f=0.0179
      52 -> 54         0.65004
      52 -> 57        -0.23097

 Excited State   3:   Singlet-A      3.8998 eV  317.93 nm  f=0.0147
      51 -> 53         0.19439
      52 -> 56         0.32967
      52 -> 58         0.57776

 Excited State   4:   Singlet-A      3.9193 eV  316.34 nm  f=0.0000
      52 -> 55         0.68308

 Excited State   5:   Singlet-A      3.9232 eV  316.02 nm  f=0.0276
      52 -> 54         0.21966
      52 -> 57         0.65123
```

TTF–CF_3

Optimized geometry

```
 C                    0    -1.75661   1.51707   0.05699
 C                    0    -2.06113   0.21559  -0.03132
 C                    0    -3.43852  -0.33843   0.16339
 S                    0    -0.08714   1.99161  -0.19127
 S                    0    -0.75097  -0.91681  -0.417
 F                    0    -3.78506  -1.15792  -0.85356
 F                    0    -4.35498   0.64756   0.23369
 F                    0    -3.53186  -1.06354   1.29804
 C                    0     0.52926   0.3122   -0.21917
 S                    0     2.45119  -1.67257  -0.14101
 C                    0     1.83954   0.00421  -0.11542
 S                    0     3.12258   1.23432   0.05327
 C                    0     4.39363   0.06659   0.41728
 C                    0     4.09336  -1.23322   0.32951
 H                    0    -2.48165   2.2981    0.25034
 H                    0     5.36979   0.46231   0.67196
 H                    0     4.79123  -2.04399   0.50282
```

TD-DFT calculations

```
Excitation energies and oscillator strengths:

 Excited State   1:   Singlet-A      2.7637 eV  448.62 nm  f=0.0009
      68 -> 69         0.69257
 This state for optimization and/or second-order correction.
 Copying the excited state density for this state as the 1-particle
RhoCI density.

 Excited State   2:   Singlet-A      3.2003 eV  387.41 nm  f=0.0203
      68 -> 70         0.67392
      68 -> 73         0.11717

 Excited State   3:   Singlet-A      3.8349 eV  323.30 nm  f=0.0014
      68 -> 70         0.10970
      68 -> 71         0.57886
      68 -> 72        -0.17355
      68 -> 73        -0.30536

 Excited State   4:   Singlet-A      3.9706 eV  312.25 nm  f=0.0337
      67 -> 69        -0.21361
      68 -> 71         0.14081
      68 -> 72         0.35061
      68 -> 73         0.16073
      68 -> 74         0.51165

 Excited State   5:   Singlet-A      4.1038 eV  302.12 nm  f=0.0270
      68 -> 71         0.28553
      68 -> 72        -0.12653
      68 -> 73         0.58092
      68 -> 74        -0.16251
```

TTF–CN

Optimized geometry

```
 C                    0     0.07531   0.09509  -0.00462
 C                    0     0.071     0.13686   1.33152
 S                    0     1.60022  -0.12576   2.17057
 C                    0     2.5799   -0.1651    0.67923
 S                    0     1.60931  -0.21778  -0.81687
 C                    0     3.93025  -0.16196   0.6801
 S                    0     4.90341  -0.09722   2.1803
 C                    0     6.3897    0.31961   1.36616
 C                    0     6.41355   0.27887   0.01707
 S                    0     4.91225  -0.22395  -0.8091
 C                    0     7.56162   0.56263  -0.76731
 N                    0     8.49506   0.79023  -1.42555
 H                    0    -0.80022   0.21542  -0.63173
 H                    0    -0.80803   0.29565   1.94498
 H                    0     7.24951   0.56959   1.97668
```

TD-DFT calculations

```
Excitation energies and oscillator strengths:

 Excited State   1:   Singlet-A      2.7043 eV  458.46 nm  f=0.0018
      58 -> 59         0.14392
      58 -> 60         0.67513
 This state for optimization and/or second-order correction.
 Copying the excited state density for this state as the 1-particle
RhoCI density.

 Excited State   2:   Singlet-A      2.7392 eV  452.63 nm  f=0.0263
      58 -> 59         0.66354
      58 -> 60        -0.14505

 Excited State   3:   Singlet-A      3.7153 eV  333.71 nm  f=0.0000
      58 -> 61         0.59940
      58 -> 63         0.32388

 Excited State   4:   Singlet-A      3.9499 eV  313.89 nm  f=0.0444
      57 -> 60         0.23427
      58 -> 62        -0.41278
      58 -> 63        -0.10919
      58 -> 64         0.45433
      58 -> 65        -0.14349

 Excited State   5:   Singlet-A      4.0841 eV  303.58 nm  f=0.0212
      58 -> 61        -0.30085
      58 -> 63         0.57610
      58 -> 64         0.18685
```

TTF–CO_2Me

Optimized geometry

```
 C                     0    -0.30472   2.52951   1.5161
 C                     0     0.76005   2.00396   0.88184
 C                     0     2.13014   2.49984   1.12797
 S                     0    -1.88951   1.91514   1.13911
 S                     0     0.44961   0.72175  -0.30801
 O                     0     2.41381   3.37973   1.91607
 O                     0     3.02908   1.84974   0.35823
 C                     0     4.39595   2.2647    0.51895
 C                     0    -1.29601   0.61472   0.0607
 S                     0    -1.50985  -1.65913  -1.49358
 C                     0    -2.10008  -0.35446  -0.42536
 S                     0    -3.84831  -0.45319  -0.06844
 C                     0    -4.06052  -2.03785  -0.81299
 C                     0    -3.01569  -2.57581  -1.45047
 H                     0    -0.21764   3.33445   2.2367
 H                     0     4.72112   2.11133   1.55101
 H                     0     4.50595   3.3213    0.26187
 H                     0     4.97138   1.6398   -0.1639
 H                     0    -5.04098  -2.49279  -0.73527
 H                     0    -3.02815  -3.53028  -1.96355
```

TD-DFT calculations

```
Excitation energies and oscillator strengths:

 Excited State   1:   Singlet-A      2.6315 eV  471.16 nm  f=0.0390
      67 -> 68         0.67844
 This state for optimization and/or second-order correction.
 Copying the excited state density for this state as the 1-particle
RhoCI density.

 Excited State   2:   Singlet-A      2.7502 eV  450.83 nm  f=0.0001
      67 -> 69         0.69394

 Excited State   3:   Singlet-A      3.6607 eV  338.69 nm  f=0.0027
      67 -> 70         0.59596
      67 -> 72        -0.33253

 Excited State   4:   Singlet-A      3.9442 eV  314.35 nm  f=0.0189
      66 -> 69        -0.14978
      67 -> 70         0.25124
      67 -> 71         0.25532
      67 -> 72         0.48644
      67 -> 73        -0.29687

 Excited State   5:   Singlet-A      4.0397 eV  306.91 nm  f=0.0216
      66 -> 69         0.14195
      67 -> 70         0.22206
      67 -> 71        -0.18993
      67 -> 72         0.32929
      67 -> 73         0.50892
```
